# Volumetric changes in alveolar ridge preservation with a compromised buccal wall: a systematic review and meta-analysis

**DOI:** 10.4317/medoral.23451

**Published:** 2020-07-19

**Authors:** Susana García-González, Andrea Galve-Huertas, Samir Aboul-Hosn Centenero, Santiago Mareque-Bueno, Marta Satorres-Nieto, Federico Hernández-Alfaro

**Affiliations:** 1 DDS, MSc, PhD. Assistant Professor of the International Master in Oral Surgery (IMOS). International University of Catalonia; 2 DDS. Resident of the International Master in Oral Surgery (IMOS). International University of Catalonia; 3 MD, PhD, EBOMFS. Co-director of the International Master in Oral Surgery (IMOS). International University of Catalonia; 4 DDS, MSc, PhD. Professor of the Master in Periodontics, University of Santiago de Compostela. University of Santiago de Compostela; 5 DDS, MS, PhD. Associate Professor, International Master’s Degree in Oral Surgery (IMOS). International University of Catalonia; 6 MD, DDS, PhD, EBOMFS. Professor and Chairman of the Department of Maxillofacial Surgery. International University of Catalonia

## Abstract

**Background:**

Many studies have addressed socket preservation, though fewer publications considering buccal wall loss can be found, since the literature typically considers sockets with four walls. A systematic review was made on the influence of type II buccal bone defects, according to Elian’s Classification, in socket grafting materials upon volumetric changes in width and height.

**Material and Methods:**

An electronic and manual literature search was conducted in accordance to PRISMA statement. The search strategy was restricted to randomized controlled trials (RCTs) and controlled clinical trials (CCTs) describing post-extraction sockets with loss of buccal wall in which alveolar ridge preservation (ARP) was carried out in the test group and spontaneous healing of the socket (SH) was considered in the control group.

**Results:**

The search strategy yielded 7 studies. The meta-analysis showed an additional bone loss of 2.37 mm in width (*p* > 0.001) and of 1.10 mm in height (*p* > 0.001) in the absence of ARP. The reconstruction of the vestibular wall was not evaluated in any study. The results also showed moderate to great heterogeneity among the included studies in terms of the changes in width and height.

**Conclusions:**

Despite the heterogeneity of the included studies, the results indicate a benefit of ARP versus SH. Further studies are needed to determine the volumetric changes that occur when performing ARP in the presence of a buccal bone wall defect.

** Key words:**Alveolar ridge preservation, buccal wall defect, volumetric changes, bone loss, meta-analysis.

## Introduction

Tooth extraction is one of the most widely performed dental procedures. Studies have demonstrated that there are marked dimensional changes of the alveolar ridge in the first 2-3 months post-extraction, and that the bone changes are more pronounced on the buccal part (1). Specifically, vertical resorption of the bone crest was found to be most marked on the facial aspect, reaching 1.4 ± 1.94 mm at 8 weeks (1). The overall decrease in width of the horizontal ridge was recorded to be approximately 50%, and two-thirds of this decrease occurred during the first three months of healing (2).

In 2005, Araújo and Lindhe conducted an experimental study in dogs to assess the dimensional alterations of the alveolar ridge occurring after tooth extraction, as well as the bone modeling and remodeling processes associated with such changes. In the first week, soft tissues were seen to cover the socket, and the coronal side presented a large amount of provisional matrix followed by a blood clot and minor reabsorption of the buccal wall. After two weeks, osteoclasts accumulated over the bone peaks of both the lingual and vestibular cortical component, and at four weeks the buccal bone exhibited more reabsorption than the lingual part. Finally, at 8 weeks the marginal portion of the buccal wall was about 2 mm apical from the marginal termination of the lingual wall (3).

The reason why reabsorption is greater at the buccal part could be catabolic changes initiated by reabsorption of the bundle bone lining the extraction socket (4). Histologically the buccal and lingual wall contains a large number of bone marrow spaces and the inner surfaces of the socket walls are lined with bundle bone. This bone is lamellar measuring 0.2-0.4 mm in thickness, and is a tooth-dependent structure. A severed periodontal ligament that included fibroblasts, distinctly orientated collagen fibers, vascular structures and inflammatory cells resided lateral to the bundle bone (3,5). When extraction is performed, the blood supply from the periodontal ligament is disrupted, and this leads to significant osteoclastic activity. In this context, as a greater proportion of the buccal plate is composed of bundle bone compared with the lingual plate, it is quickly reabsorbed (4). This phenomenon is also attribute to the limited thickness of the facial bone wall in comparison with the lingual/palatal aspect of the socket (6). In 2018 López-Jarana *et al*. anatomically described the bone morphology in the maxillary and mandibular tooth areas using cone-beam computed tomography (CBCT); the thickness of the buccal plate was less than 2mm in over 80% of the anterior and posterior teeth, in the maxilla the most critical areas were laterals incisors, canines and first premolars and in the mandible central incisors, lateral incisors and canines (7).

As a result of the above, there is growing interest in avoiding and regenerating such reabsorption of bone following tooth extraction. The main preventive strategy is socket preservation (SP), which involves grafting (filling and augmenting) an intact socket. The aim of SP is to fully preserve the vertical and horizontal dimensions. A socket failing to meet these conditions is referred to as a damaged socket and can be classified as 3-, 2- or 1-walled, or lack of vertical height at all four walls, or exhibit apical fenestration (8). An additional socket classification is Elian’s that consists in three types: a) Type I socket: the facial soft tissue and buccal plate of the bone are at normal levels in relation to the cementoenamel junction of the pre-extracted tooth and remain intact post-extraction. Those sockets are the easiest and most predicTable to treat and the ideal treatment is an immediate implant; b) Type II socket: facial soft tissue is present but the buccal plate is partially missing following extraction of the tooth. These sockets are sometimes treated as Type I socket leading to a less than ideal aesthetic results and a safer treatment is socket preservation, therefore is a socket that must be carefully evaluated before any treatment; c) Type III socket: The facial soft tissue and the buccal plate of bone are both markedly reduced after tooth extraction. Sockets in this classification require very high dental experience, skills and time consuming for success. The ideal treatment option is the extraction, wait two months and perform a bone regeneration and afterword place the implant (9).

Within type II socket the option of grafting a damaged socket is called alveolar ridge preservation (ARP), which seeks not only to preserve the bone quantity present at extraction, but also to regain previously missing structures (8).

Many studies have addressed socket preservation, though fewer publications considering buccal wall loss can be found. These dimensional changes are important for decision-making and comprehensive treatment planning, particularly when there is lack of buccal wall.

The present study offers a systematic review of the scientific evidence on the influence of type II buccal bone defects, according to Elian’s classification (9), in socket grafting materials upon volumetric changes in width and height, compared to sockets with spontaneous healing.

## Material and Methods

- Search strategy and focused question

Electronic and manual literature searches were conducted by two independent reviewers (S.G-G and A.G-H.) in the National Library of Medicine (Medline via PubMed) for articles published up until February 2020.

The proposed methods were registered with PROSPERO (CRD42018115043) and based on the PICO (participant, intervention, comparison, outcome) model - the main question of the study being: Is there any scientific evidence on the influence of type II buccal bone defects in socket grafting materials upon changes in width and height?

With the purpose of avoiding risk of bias, this review was based on the Preferred Reporting Items of Systematic Reviews and Meta-Analyses (PRISMA), (10) comprising a 27-item checklist and a four-phase flow chart.

The search terms used were MeSH keywords and other free terms. Boolean operators (OR, AND) were used to combine searches: ((alveolar[All Fields] AND socket[All Fields]) OR (socket[All Fields] AND ("preservation, biological"[MeSH Terms] OR ("preservation"[All Fields] AND "biological"[All Fields]) OR "biological preservation"[All Fields] OR "preservation"[All Fields])) OR (ridge[All Fields] AND ("preservation, biological"[MeSH Terms] OR ("preservation"[All Fields] AND "biological"[All Fields]) OR "biological preservation"[All Fields] OR "preservation"[All Fields] OR "socket preservation"[All Fields] OR "alveolar ridge augmentation"[MeSH Terms]))) AND (histomorphometric[All Fields] OR (new[All Fields] AND ("osteogenesis"[MeSH Terms] OR "osteogenesis"[All Fields] OR ("bone"[All Fields] AND "formation"[All Fields]) OR "bone formation"[All Fields])) OR histologic[All Fields]) AND ((buccal[All Fields] AND defect[All Fields]) OR (wall[All Fields] AND defect[All Fields]) OR ("bone diseases, metabolic"[MeSH Terms] OR ("bone"[All Fields] AND "diseases"[All Fields] AND "metabolic"[All Fields]) OR "metabolic bone diseases"[All Fields] OR ("bone"[All Fields] AND "loss"[All Fields]) OR "bone loss"[All Fields]) OR defect[All Fields] OR (three[All Fields] AND wall[All Fields])).

- Study selection criteria

The inclusion criteria were as follows: 1) no language restriction; 2) randomized controlled trials (RCTs); 3) controlled clinical trials (CCTs); 4) studies describing sockets with buccal wall loss; 5) studies describing the use of biomaterials and/or barriers; 6) studies with test groups involving graft-based alveolar ridge preservation (ARP); and 7) studies with controls involving spontaneous healing (SH) or placement of a collagen membrane (CM).

The exclusion criteria were as follows: 1) unclear information on the patient, study design or follow-up; 2) the lack of a control group; 3) a mean follow-up of under two months or an unspecified duration of follow-up; 4) failure to specify the number of patients and teeth subjected to socket preservation; and 5) no accurate description of facial wall bone loss.

- Screening process

First, two reviewers S.G-G and A.G-H. screened the titles and abstracts independently and performed the primary search. The studies appearing to meet the inclusion criteria, or those with insufficient data in the title and abstract to make a clear decision, were then retrieved for full-text evaluation, which was carried out independently by the same two reviewers. Any disagreement was resolved by discussion with two other reviewers (S.A-G and S.M-B).

The reasons for rejecting studies at this or at subsequent stages were recorded. Special attention was paid to duplicate publications, in order to avoid a likely greater impact of the same data upon the overall results.

- Data analysis

The primary outcome of the meta-analysis was the dimensional changes of the socket in terms of width and/or height with ARP and control sites. The change or variation in bone (final versus baseline) was taken to be negative in the event of bone loss (referred to either width or height).

The weighted mean differences (WMDs) were the measure of global effect, estimated by a meta-analysis of random effects with a test based on 95% confidence intervals (95%CIs).

Heterogeneity was calculated based on the I2 index (percentage of variability of the estimated effect that can be attributed to heterogeneity of the true effects) and the corresponding nullity of Q test. The consistency of the results of the different studies was explored by Galbraith plots.

Funnel plots and the Egger test were used to assess selection bias. The modified Cochrane Collaboration tool for randomized controlled trials was used to assess risk of bias. Bias was assessed as a judgment (high, low or unclear) of individual elements from 5 domains (selection, performance, attrition, reporting and other).

The level of significance used in the analyses was 5% (α = 0.05). The R 3.0.2 package was used throughout.

## Results

- Study selection

**Figure 1:**
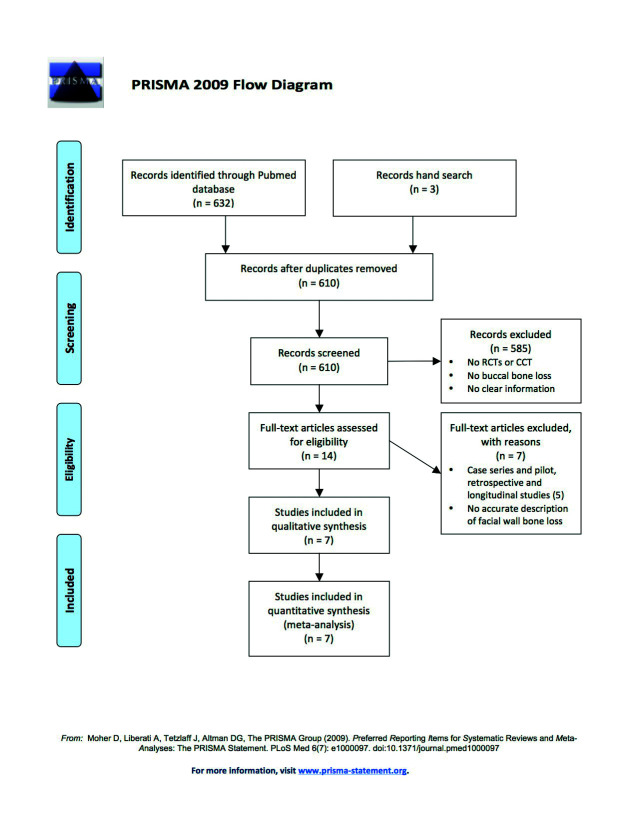
Flow chart of the screening process.

Initial screening yielded a total of 366 articles. After evaluation of the abstracts, the full texts of 15 studies were obtained and reviewed. Of these, 7 articles met the inclusion criteria and were included in the analysis (Fig. 1).

Case series and pilot, retrospective or longitudinal studies were excluded (11-16). In addition, two studies were excluded because the data referred to bone loss in the buccal wall were inadequate (17,18). All the included studies were RCTs (level of evidence 1), (19-24) with the exception of one CCT (level of evidence 1) (25). The details of the included studies are summarized in Table 1.

**Table 1:**
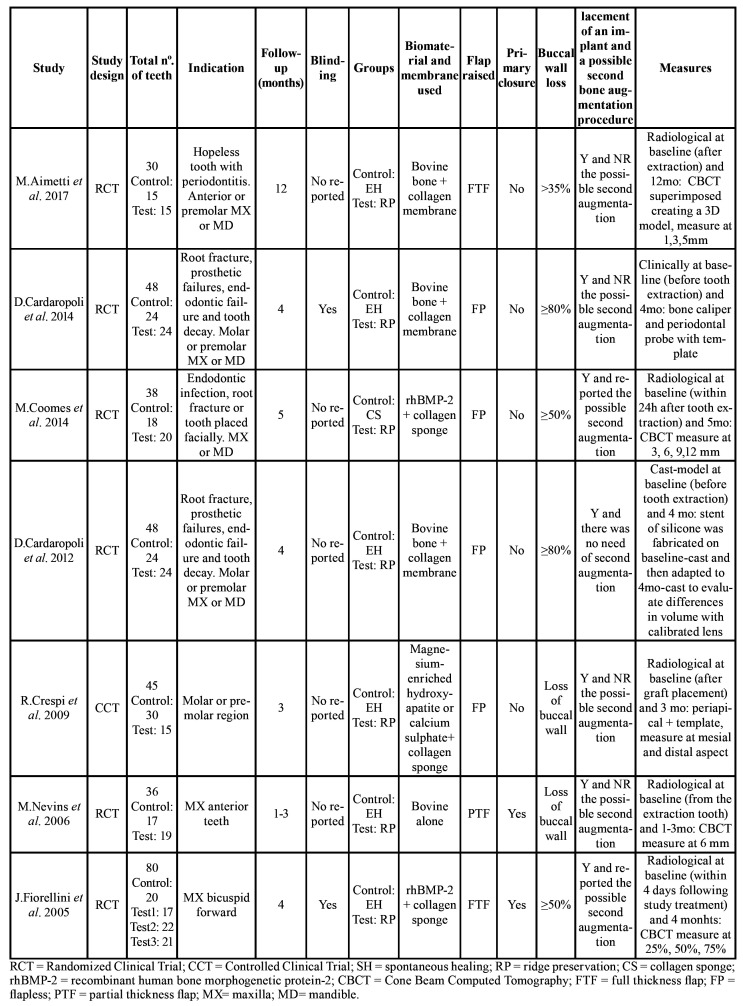
Characteristics of the studies included in the qualitative assessment.

- Characteristics of the included studies

Intervention type and sample characteristics

The studies mainly included two groups; a control group with spontaneous healing after tooth extraction (of the exception being the study of Coomes *et al*. (23), which used a collagen sponge) and a test group in which ARP was performed. There are no studies comparing sockets with spontaneous healing and the use of a collagen sponge which it liquefies after a week or less and is completely absorbed in four to six weeks, without excessive scar formation (26). However Serino *et al*. evaluate whether ARP following the application of a bioabsorbable polylactide-polyglucolide sponge and the results showed that alveolar bone resorption after tooth extraction may be prevented or reduced specially in sockets where the buccal bone is completely or partially lost (17). Two studies had more than one test group. Crespi *et al*. (25) compared split-mouth treatment involving magnesium enriched hydroxyapatite (MHA) and calcium phosphate (CP). In turn, Fiorellini *et al*. (19) compared three groups receiving 0.00 mg/ml, 0.75 mg/ml or 1.50 mg/ml rhBMP-2 with a collagen sponge (CS). Fiorellini *et al*. (19) and Coomes *et al*. (23) used rhBMP-2 and a CS for ARP; the rest of the authors performed socket augmentation using bovine bone (Bio-Oss® Collagen; Geistlich Pharma AB, Wolhusen, Switzerland) with a collagen membrane covering (Bio-Gide®; Geistlich Pharma AB).

Soft tissue management

On the test sides, the flapless technique was the most widely used approach, without primary closure, and in some cases sutures were placed. Nevins *et al*. (20) performed a partial thickness flap with primary closure but without specifying how complete wound closing was made. Aimetti *et al*. (24) and Fiorellini *et al*. (19) raised a full thickness flap. In the case of Aimetti *et al*. (24), there was no primary closure - the flap being repositioned at the presurgical level and sutured with horizontal mattress sutures. Fiorellini *et al*. (19) performed sulcular and vertical incisions with tension-free soft tissues wound closure.

Evaluation period

The follow-up period was homogenous, with an average of 4.85 months. In most of the articles, final follow-up was matched with implant placement at around 3-5 months, depending on the biomaterial used. The exception was the study of Aimetti *et al*. (24), in which the mean follow-up was 12 months.

Methods of measurements 

Bone assessment in the included articles was mostly done with CBCT. Only in the articles of Aimetti *et al*. (24) and Coomes *et al*. (23) was overlapping made of the CBCT study at baseline and at the end of the study. Crespi *et al*. (25) used periapical radiographs with an occlusal template; Cardaropoli *et al*. [2014] (22) performed a clinical estimation of the bone with calipers and a periodontal probe; and Cardaropoli *et al*. [2012] (21) assessed bone on a dental cast obtained at baseline and after four months. The authors produced a silicon stent on each baseline cast, and this stent was adapted on the four-month cast to evaluate the differences in volume using a calibrated lens.

- Changes in width

A total of 6 studies (19,21-25) provided valid information on bone changes in width. Cardaropolli *et al*. reported an average bone variation of -1.04 ± 1.96 mm in the group test and of -4.48 ± 1.52 mm in the control group - the difference being statistically significant. The rest of the studies reported similar findings (Table 2).

The meta-analysis determined a weighted mean difference of 2.37 mm, corresponding to the additional bone loss in the absence of ARP – the results being statistically significant (*p* < 0.001) (Table 3). Unfortunately the included studies measured the width changes as distances from buccal to lingual wall, no evaluation of the buccal was performed neither in the articles that evaluated the need nor not for second bone regeneration when implant was placed (19,21,23).

**Figure 2:**
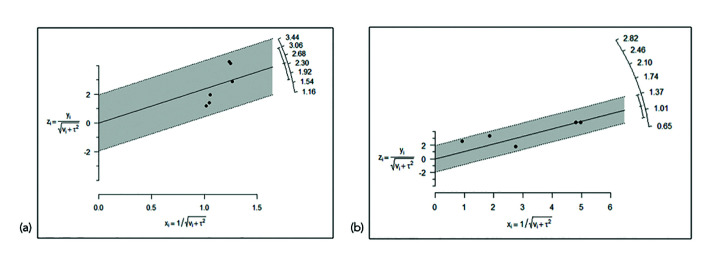
Galbraith plot of the homogeneity of the studies referred to (a) changes in width; and (b) changes in height.

The heterogeneity of the 6 included studies was I2 = 85.9%, and the Cochran Q test concluded that homogeneity can not be accepted. Nevertheless, the Galbraith plot (Fig. 2) showed accepTable consistency between the different studies. No concrete study was responsible for the observed heterogeneity. In this regard, although the degree of heterogeneity was high, the results of the meta-analysis were not invalidated as a result, since all the studies pointed in the same direction with similar sample size and/or variability.

**Figure 3:**
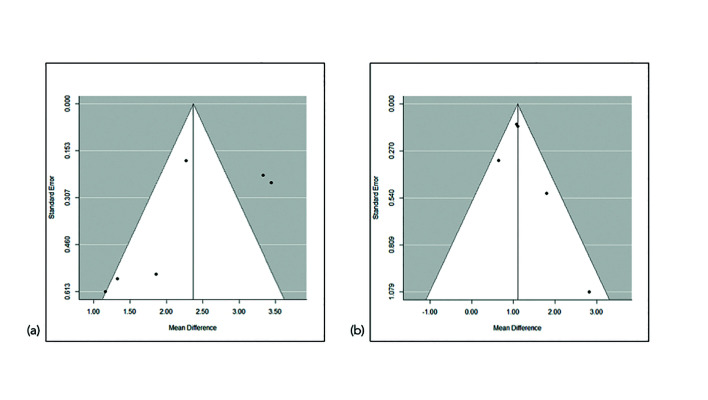
Meta-analysis funnel plots showing the risk of bias referred to (a) changes in width; and (b) changes in height.

**Figure 4:**
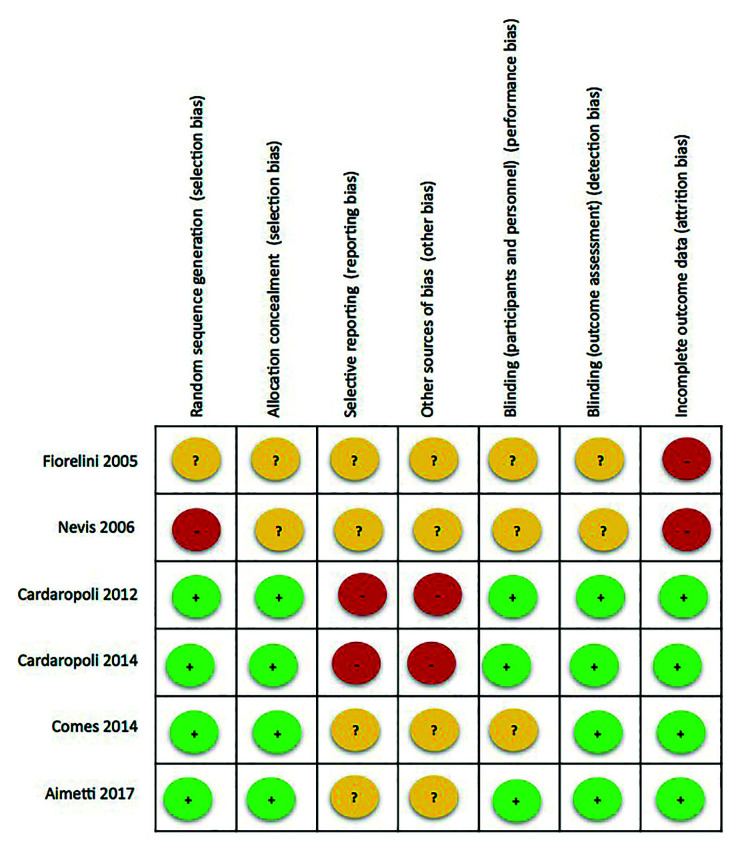
Quality assessment of the included studies: risk of bias summary. + = low risk of bias; - = high risk of bias; ? = unclear risk of bias.

Publication bias was analyzed by funnel plots (Fig. 3). Despite the limited statistical power attribuTable to the small number of articles, the Egger test revealed significant asymmetry (*p* = 0.038). The assessment of study quality was performed for all the included papers; the Cochrane Collaboration’s tool for assessing risk of bias was used in the case of randomized controlled clinical trials and controlled clinical trials (Fig. 4).

**Table 2:**
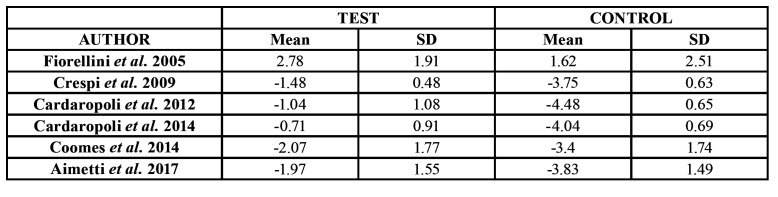
Changes in bone width (mean ± standard deviation [SD]).

**Table 3:**
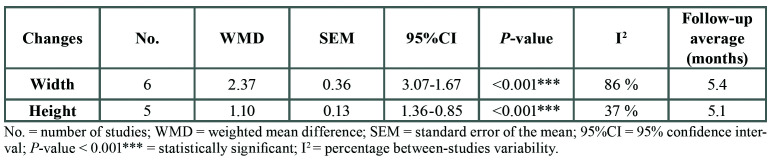
Results of the meta-analysis referred to changes in bone height and width.

- Changes in height

A total of 5 studies (19-22,24) provided valid information on bone changes in height. All studies reported greater loss in the control group. Aimetti *et al*. even reported bone gain in the treatment group (Table 4).

The meta-analysis revealed a weighted mean difference of 1.10 mm between groups. Again, 1.10 mm represented the bone preserved thanks to the ARP. The result proved statistically significant (*p* < 0.001) (Table 3).

There was an accepTable degree of homogeneity among the 5 studies, as suggested by I2 = 37.2%. The Galbraith plot (Fig. 2) likewise showed the 5 studies to fall within the confidence range, thus warranting the consistency of the results obtained.

Regarding possible publication bias, the study conducted by Nevins *et al*. was imprecise in that it reported a very large effect size and standard deviation, as seen in the funnel plot (Fig. 3). In any case, the Egger test had only limited power (*p* = 0.171) in seeking to detect significant bias. In contrast, the remaining four studies exhibited a symmetrical conFiguration.

**Table 4:**
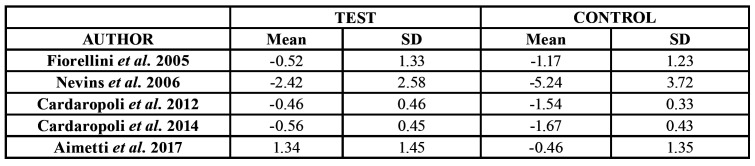
Changes in height (mean ± standard deviation [SD]).

## Discussion

- Main findings

Following an exhaustive literature search, 7 studies were finally identified: 6 RCTs (19,24) and a single CCT (25). The rest of the studies were excluded due to a lack of scientific evidence, and because the evaluation of ARP requires the comparison of a test group in which bone grafting is performed versus a control group involving either spontaneous healing or the placement a collagen sponge.

Despite the heterogeneity of the studies, which was found to be high in relation to changes in width and moderate in changes in height, all the articles showed the same patterns and reached the same conclusions: ARP significantly reduces bone loss compared to the controls in post-extraction sockets with vestibular wall loss. Similar findings moreover applied to intact sockets, and authors such as Avila-Ortiz *et al*., (27) Tomlin *et al*. (28) or Jambhekar *et al*. (29) agreed that ARP is effective in limiting bone crest reduction in the context of a single extraction.

In this study we recorded an additional loss of 2.37 mm in width and 1.10 mm in height in the absence of ARP (*p* < 0.001). Bone loss was greater in width than in height, in the same way as is also seen in sockets with four walls. In 2011, Vignoletti (30) carried out a systematic review and recorded greater dimensional changes in width in the control group than in the test group in which socket preservation was performed.

In a type II socket (with buccal dehiscence) in the anterior area, the clinician has several treatment options: 1) ARP: there are different ridge preservation techniques following tooth extraction, including placement of different graft materials and/or use of occlusive membranes to cover the socket entrance. Grafts are generally classified according to their original sources: autograft, allograft, xenograft and alloplasts. The alloplastic or synthetic biomaterials are inert and osteoconductive filler materials, which serves as a scaffold for new bone formation (31). The sponges made of collagen or polylactic/polyglycolic acid are a common example of synthetic bone graft material. Lekovic *et al*. in 1998 reported that the healing of extraction sites seems to occur with different degrees of bone resorption, which can be partly prevented by the use of resorbable membranes made of glycolide and lactide polymers (32). For that reason, Serino *et al*. in 2002 evaluated the application of a bioabsorbable polylactide and polyglycolide sponge, which prevented or reduced the bone resorption following tooth extraction and the quality of bone form was optimal for dental implant insertion (17). Further articles assessed the effectiveness of this biomaterial, Ohba *et al*. explains the advantages of this sponge-like characteristics on ARP, such as: good handling, easy trimming, tight contact with surrounding bone of the extraction socket by swelling and increasing stability by swelling (33). A recent article Covani *et al*. 2020, defends the use of collagen right after tooth extraction to sustain the soft tissue and provide initial structural and mechanical support to the healing tissues, releasing the tension in the most coronal part of the wound (34). 2) Early implant placement: Buser *et al*. proposed the use of it combined with flap surgery, guided bone regeneration and submerged healing after soft tissue healing (35). This method requires three operations, namely, tooth extraction, implant insertion and soft tissue plasty 3) Immediate implant with guided bone regeneration: Although it has always been described that fully intact facial bone wall at the extraction site is the most important requirement for immediate implant placement (35), however a study using CBCT have shown that a thick bone wall phenotype is rarely present in the anterior maxilla, with approximately 4.6% of patients having a thick wall phenotype in the central incisor area (>1mm) (36). Implant placement for a single anterior maxillary tooth with a facial bone wall defect are rarely reported, the most recent article reported is from Liu *et al*. in 2019, a prospective study with only forty-five patients treated with flap surgery, guided bone regeneration and non-submerged healing. Even thought they found good results, the study has limitations and long-term studies are needed to confirm this technique (37). Moreover in the literature we find more articles related to this technique but they are case series or case reports (38,39).

- Implant placement and alveolar ridge preservation

Tooth extraction is sometimes followed by severe bone deficiencies that pose a challenge for clinicians since in some cases implant placement simultaneous to bone augmentation is feasible, while in other cases it is not. The decision of whether or not primary bone augmentation is needed depends on a number of factors, such as primary stability, correct placement of the implant in a prosthetically guided position, or whether the expected extent of exposure of the implant surface is predictive of bone regeneration (35). No plausible justification can be found in the included studies as to why ARP was performed instead of placing the implant with lateral augmentation. On the other hand, however, dehiscence presented by the included sockets exceeded 50-80%, when it is known that lateral bone augmentation with simultaneous dental implant placement is generally documented with buccal dehiscences of up to 8 mm in height. For this reason it is logical that in the included studies no implants were placed because they should have a dehiscence of 10 mm (40).

Implants were placed in all studies, and only the studies of Coomes *et al*. (23), Cardaropoli *et al*. 2012 (21) and Fiorellini *et al*. (19) mentioned the need or not for second bone regeneration. In the publication by Cardaropoli *et al*. 2012 (21), ARP allowed implant placement without the need for bone augmentation. On the other hand, Fiorellini *et al*. (19) noted that in those cases where rhBMP-2 > 1.50 mg/ml was placed, there were significantly fewer procedures compared to no treatment or the application of 0.75 mg/ml. The same occurred in the study of Coomes *et al*. (23), where secondary ridge augmentation proved necessary as a separate surgical procedure in one patient in the test group compared to 6 patients in the control group. For this reason, ARP added no further complexity to the surgical procedure other than tooth extraction.

- Heterogeneity, meta-analysis and publication bias

In the case of changes in bone width, in which 6 articles were included, heterogeneity was quantified as I2= 85.9%. The Cochran Q test logically concluded that homogeneity cannot be accepted. The two articles published by Cardaropoli *et al*. (21,22) were those reporting the greatest benefit with the regeneration technique, while the remaining four articles (19,23,25) showed no overlap with those of Cardaropoli *et al*. (21,22) in the confidence interval. One strategy for addressing the problem of heterogeneity is to investigate the methods, design and population of the two groups of studies. The evidence indicates that the two studies carried out by Cardaropoli *et al*. (21-22) obtained such favorable results due to some feature distinguishing them from the other four articles (19,23,25). Fiorellin *et al*. (19) published the only study reporting bone gain in both groups, albeit with a comparatively greater gain in the test group. These observations were not heterogeneous with respect to Coomes *et al*. (23) or Aimetti *et al*. (24). The consistency of the outcomes of the different studies according to their accuracy was also explored. All of them presented similar precision because they had a similar sample size and/or similar variability. In this sense, the Galbraith plot (Fig. 2) showed accepTable consistency between the different studies in the sample. No concrete study was responsible for the observed heterogeneity, though the two articles published by Cardaropoli *et al*. (21,22) contributed to practically the same degree as the other four (19,23,25). Thus, although the degree of heterogeneity was high, the findings of the meta-analysis are not invalidated as a result: since all the studies point in the same direction, a clear common effect may be postulated (benefit of regeneration), though of variable magnitude depending on the study. Publication bias referred to the changes in width was analyzed from the Funnel plots (Fig. 3). Despite the limited statistical power attribuTable to the small number of articles, the Egger test appointed to significant asymmetry (*p* = 0.038). This was evidenced by the Funnel plots (Fig. 3), with three studies at the lower left, exhibiting high standard error (upward imprecision due to excessive variability, small sample size or both), and reporting moderate benefit of treatment. On the other hand, the two studies at the upper right proved more accurate and reported important benefit of treatment. In sum, there were no imprecise studies concluding great advantages of the treatment, and the test conclusions referred to benefit were strengthened as the studies proved more rigorous. In this sense, the observed asymmetry likewise does not invalidate the results of the meta-analysis.

In the case of changes in bone height, the recorded value I2=37.2% suggests accepTable homogeneity of the 5 included studies. The Galbraith plot (Fig. 2) also showed the 5 studies to lie within the confidence interval, thus warranting the consistency of the results obtained. Regarding possible publication bias, it should be noted that the study of Nevins *et al*. (20) is positioned at the lower right end of the funnel plot (Fig. 3) due to two main reasons. Firstly, it reports a very large effect size, and thus the difference in bone loss between the test group and the controls is very large (2.82 mm), with a great standard deviation of the pre-post difference in bone dimension. Secondly, this was an imprecise study reporting a great advantage of ARP, and the fact that there were no published studies of similar precision reporting contradictory or more moderate results is striking and questions the absence of bias. In any case, since only 5 articles were included in reference to the changes in bone width, the power of the Egger test was limited (*p* = 0.171) in attempting to identify such bias as being significant. With the exception of the study by Nevins *et al*., (20) the other four publications (19,21,22,24) did exhibit a symmetrical conFiguration.

- Limitations

The main limitation of our study is the lack of literature on the subject. Although many studies on ARP have been published, they characteristically contemplate sockets with four walls - despite the fact that 53% of all post-extraction sockets have dehiscences or fenestrations (4). It is therefore very common to find sockets with a loss of buccal wall, though our systematic review only found 7 studies that take this fact into account. Another limitation is the lack of standardization among the different studies referred to the bone regeneration methods used; the study variables considered (clinical or radiological); the use of full or partial thickness flaps; and the size of the buccal bone dehiscence involved. All this must be taken into account on interpreting the results obtained. On the other hand, a comparison could have been made between sockets with three and four walls, in order to assess the differences in terms of bone loss. It also would have been interesting to include histological results of biopsies, assessing percentage bone formation, residual particles and connective tissue. This was not done in our case because only three of the 7 articles had performed biopsies at the time of implant placement, and all three differed in terms of the biomaterial used. Lastly, by focusing on CCTs and RCTs, with the exclusion of other types of studies, significant information may have been omitted, and this could have conditioned the results obtained.

- Future lines of research

Although socket preservation is a well-studied technique, further clinical studies are needed to investigate dehiscence and fenestration present after extraction and their influence upon the dimensional changes in ARP. Additional and more homogeneous studies involving a follow-up period of 5 years are needed to assess the success and survival of implants placed in ARP with buccal dehiscences. It also would be interesting to study the biological behavior of the different biomaterials and membranes found on the market. With the new developments in tissue engineering, another interesting field of research would be the application of different growth factors within the post-extraction socket.

## Conclusions

Alveolar ridge preservation significantly reduced the loss of bone width by 2.37 mm compared with the control group. This result was obtained under conditions of great heterogeneity among the 6 studies included in the meta-analysis, though all of them agreed in evidencing a benefit of treatment.

Similar results were obtained regarding the changes in bone height, with regeneration avoiding 1.10 mm of bone loss. This likewise proved statistically significant. In this case, the heterogeneity of the 5 studies included in the meta-analysis was only moderate.

Although ARP is a widely studied technique, further research is needed, taking into account the possibility of post-extraction socket fenestration or dehiscence.
